# Cell Uptake and Validation of Novel PECs for Biomedical Applications

**DOI:** 10.1155/2011/203676

**Published:** 2011-08-25

**Authors:** Ilaria E. Palamà, Mariarosaria Musarò, Addolorata M. L. Coluccia, Stefania D'Amone, Giuseppe Gigli

**Affiliations:** ^1^NNL, Institute of Nanoscience CNR, Street Arnesano, 73100 Lecce, Italy; ^2^Scuola Superiore ISUFI, University of Salento, Street Arnesano, 73100 Lecce, Italy; ^3^Hematology and Clinical Proteomics Unit, “Vito Fazzi” Hospital, University of Salento, Square Muratore, 73100 Lecce, Italy; ^4^Dipartimento Ingegneria dell'Innovazione, University of Salento, Street Monteroni, 73100 Lecce, Italy; ^5^Center of Biomolecular Nanotechnologies (CNB) of Italian Institute of Technology (IIT), Street Barsanti, 73010 Arnesano (Le), Italy

## Abstract

This pilot study provides the proof of principle for biomedical application of novel polyelectrolyte complexes (PECs) obtained *via* electrostatic interactions between dextran sulphate (DXS) and poly(allylamine hydrochloride) (PAH). Scanning electron microscopy (SEM) and atomic force microscopy (AFM) showed that DXS/PAH polyelectrolyte complexes were Monodispersed with regular rounded-shape features and average diameters of 250 nm at 2 : 1 weight ratios of DXS/PAH. Fluorescently labelled DXS and fluorescein-isothiocyanate- (FITC-)conjugate DXS were used to follow cell uptake efficiency of PECs and biodegradability of their enzymatically degradable DXS-layers by using confocal laser scanning microscopy (CLSM). Moreover, quantitative MTT and Trypan Blue assays were employed to validate PECs as feasible and safe nanoscaled carriers at single-cell level without adverse effects on metabolism and viability.

## 1. Introduction

Polymeric particles have sparked major interest as nanosized delivery systems that increase drug's retention effects and efficacy at single-cell level through a temporal or spatial control (*targeted therapy*) [1–3]. From a biological and therapeutic perspective, the intrinsic advantage of these supramolecular assemblies lies in the potential of acting as biocompatible and biodegradable carriers under physiological conditions [4–6]. Indeed, the use of polymers that are biodegradable or can release their cargo in response to well-defined stimuli, enables small molecule drugs to be delivered over an extended period of time, thus achieving effective therapeutic effects at reduced drug concentrations or dosage frequency [7–9]. Due to their versatility in templating and surface coating, several polymeric nanosystems such as polyelectrolyte capsules [10–12], polyionic complexes (PICs), or polyelectrolyte complexes (PECs) are being largely characterized by addressing their uptake kinetics and processing in living human cells [13–16]. 

We herein describe a simple and reproducible procedure to obtain the spontaneous formation of polyelectrolyte complexes (PECs) by mixing oppositely charged polyions, such as poly(allylamine hydrochloride) (PAH) and dextran sulphate (DXS). The electrostatic interactions between such polycations and polyanions in solution can be finely tuned in the nanometer range by varying the strength and location of ionic sites, polymer chain rigidity, precursor chemistries, pH, temperature, and mixing intensity [15, 16]. The physiochemical properties of PECs were evaluated by the measurement of particle size, *ζ* potential, atomic force microscopy (AFM), and scanning electron microscopy (SEM), showing that DXS/PAH polyelectrolyte complexes were monodispersed with regular rounded-shape features and average diameters of 250 nm. PAH was chosen as it is a typical example of synthetic polycation that is insensitive to the action of intracellular proteases, whereas the polyanion DXS contains protease-sensitive bonds that ensure the polymer's erosion inside the cells. Moreover, confocal laser scanning microscopy (CLSM) and several functional approaches were employed to validate PECs as biodegradable nanoscaled carriers at single-cell level without adverse side effects on cell metabolism and viability.

## 2. Materials and Methods

### 2.1. Chemicals

The sources of the chemicals are as follows: poly(allylamine hydrochloride) (PAH, Sigma, USA, CAS:71550-12-4), dextran sulfate sodium salt from Leuconostoc sppe (DXS, Sigma, USA, CAS:077K1027), Fluorescein isothiocyanate dextran (DXS-FITC, Sigma, USA, CAS: 20696KJ), formaldehyde solution (Sigma, USA, CAS:50-00-0).

All tissue culture media and serum were purchased from Sigma, cell lines were purchased from American Tissue Type Collection (ATTC). The suppliers of the chemicals were as follows: fetal bovine serum (FBS, Sigma, USA, CAS:N/A), penicillin-streptomycin solution (Sigma, USA, CAS:010M0651), L-glutamine 200 mM (Sigma, USA, CAS:56-85-9), DMEM medium (Sigma, USA, CAS:N/A), sodium pyruvate (Sigma, USA, CAS:113-24-6), Trypsin-EDTA solution (Sigma, USA, CAS:N/A), thiazolyl blue tetrazolium bromide (MTT, Sigma, USA, CAS:MKBB9557), phosphate buffered saline (PBS, Sigma, USA, CAS:049K8204), Trypan Blue solution (Sigma, USA, CAS:72-57-1), Fluoroshield with DAPI (Sigma, USA, CAS:070M1418).

### 2.2. Synthesis of Polyelectrolyte Complexes (PECs)

Polyelectrolyte complexes (PECs) were prepared according to Cheng et al., 2009 [15]. Solution of DXS-FITC (2 mg/mL in 0.1 M NaCl pH 6.5) was mixed with a solution of PAH (1 mg/mL in 0.5 M NaCl pH 6.5) under agitation and the mixture solution was stirred slowly at room temperature for 2 hours. After this period, the solution was dried in a lyophilisator and stored at 4°C.

### 2.3. Polyelectrolyte Complexes Size and *ζ* Potential Measurement

Dynamic light scattering (DLS) and zeta-potential (*ζ*) measurements were performed on a Zetasizer Nano ZS90 (Malvern, USA) equipped with a 4.0 mW He-Ne laser operating at 633 nm and an avalanche photodiode detector. Measurements were made at 25°C in aqueous solutions (pH 7). The PECs solution (1 mg/mL) was passed through a 0.45 *μ*m pore size filter before measurements and appropriately diluted if necessary according to the instrument's requirements. Representative measurements of three distinct sets of data have been reported (Student* t*- test, *P* < 0.05).

### 2.4. Scanning Electron Microscopy (SEM)

For SEM analysis (RHAIT 150), samples were prepared by applying a drop of the particle suspension to a SiO_2_ wafer and then drying overnight. Prior to SEM observation, the samples were sputter-coated with a 10 nm gold layer to make them electronically conductive and to avoid electronic charging during SEM imaging.

### 2.5. Atomic Force Microscopy (AFM)

The morphological characterization has been performed by tapping mode AFM using a Solver PRO Scanning Probe Microscope (NT-MDT) in air at room temperature, we used TESPA (Veeco, USA) silicon cantilevers of 20–80 N/m spring constant and resonance frequency of around 300 kHz. A drop of sample suspension was applied to a silicon support.

### 2.6. Cell Culture

Human fibroblast cell line (BJ) and human cervix carcinoma cell line (HeLa) were maintained in DMEM supplemented with FBS (10%), penicillin (100 U/mL culture medium), streptomycin (100 *μ*g/mL culture medium), L-glutamine (5%), and sodium pyruvate (5%). Cells were grown in a humidified incubator at 37°C, 5% CO_2_, and 95% relative humidity.

### 2.7. PECs Uptake by HeLa and BJ Cells

#### 2.7.1. Confocal Laser Scanning Microscopy (CLSM)

To determine the cellular uptake of the PECs we seeded 10^5^ cells/mL in sterile glass-culture slide. The cells were incubated with the PECs dispersions at a concentration of 0.05 mg/mL. After 3 hours of incubation at 37°C, the culture medium was removed, and the cells were washed three times with phosphate buffered saline. For fluorescent microscopic observation, cells were fixed in situ for 5 minutes in 3.7% formaldehyde and mounting with fluoroshield with DAPI. Confocal micrographs were taken with Leica confocal scanning system mounted into a Leica TCS SP5 (Leica Microsystem GmbH, Mannheim, Germany), equipped with a 63  X oil immersion objective and spatial resolution of approximately 200 nm in *x*-*y* and 100 nm in *z*. The 3D confocal scanning is performed by reconstructing the photoluminescence coming from different focalized slices with a sequential image acquisition. The optical sections were collected in transverse *x*-*z* and *y*-*z* planes.

### 2.8. Cytotoxicity Analysis

#### 2.8.1. MTT Assay

The MTT system is a reproducible means of measuring the activity of living cells via mitochondrial dehydrogenase activity. The key component is 3-[4,5-dimethylthiazol-2-yl]-2,5-diphenyl tetrazolium bromide. Mitochondrial dehydrogenases of viable cells cleave the tetrazolium ring, yielding purple MTT formazan crystals that are insoluble in aqueous solutions. The crystals can be dissolved in acidified isopropanol. The resulting purple solution is spectrophotometrically measured. An increase in cell number results in an increase in the amount of MTT formazan formed and an increase in absorbance. The polyelectrolyte complexes suspension at 0.01, 0.03, 0.05, 0.1, 0.2 mg/mL concentrations was diluted with appropriate cultural medium. The MTT method of cell determination is most useful when cultures are prepared in multiwell plates. HeLa and BJ cells (10^5^ cells/mL) were added to 24-well culture plates at 1000 *μ*L/well and incubated at 37°C in 5% CO_2_, 95% relative humidity for 24–48 and 72 hours with the PECs suspension. The control was complete culture medium. After an appropriate incubation period, remove cultures from incubator and aseptically add MTT solution in an amount equal to 10% of the culture volume. Return cultures to incubator and incubate for 3 hours. After the incubation period, remove cultures from incubator and dissolve the resulting MTT formazan crystals with acidified isopropanol solution an equal culture volume. The plates should be read within 1 hour after adding acidified isopropanol solution. After the incubation time pipetting up and down may be required to completely dissolve the MTT formazan crystals. Spectrophotometrically measure absorbance a wavelength of 570 nm. Subtract background absorbance measured at 690 nm.

The percentage viability was expressed as the relative growth rate (RGR) by equation: RGR = (D_sample_/D_control_) ∗ 100% where D_sample_ and D_control_ are the absorbances of the sample and the negative control.

#### 2.8.2. Trypan Blue Assay

Trypan Blue is one of the dye exclusion procedures for viable cell counting. This method is based on the principle that live (viable) cells do not take up certain dyes whereas dead (nonviable) cells do. Live cells with intact cell membranes are not colored. Because cells are very selective in the compounds that pass through the membrane, in a viable cell, Trypan Blue is not absorbed; however, it traverses the membrane in a dead cell. Therefore, dead cells are shown to be a distinctive blue color under a microscope, and living cells are excluded from staining. For the trypan blue assay, HeLa and BJ cells were added to 24-well culture plates at 1000 *μ*L/well and incubated at 37°C in 5% CO_2_, 95% relative humidity for 24, 48, and 72 hours with PECs suspension. The polyelectrolyte complexes suspension at 0.01, 0.03, 0.05, 0.1, 0.2 mg/mL concentrations was diluted with culture medium. The control was complete culture medium. After the appropriate incubation period, the medium of each sample was collected. After trypsinization, the cells were collected and centrifuged. The pellet was resuspended in an appropriate amount of medium. The collected cells were mixed with the same volume of 0.4% trypan blue solution. Cells were allowed to stand from 5 to 15 min. Later, 10 *μ*L of stained cells was placed in a hemocytometer, and the number of viable (unstained) and dead (stained) cells was counted with a light microscope. The viability percentage was expressed using the following equation: cell viability (%) = total viable cells (unstained)/total cells (stained and unstained) × 100. Representative measurements of three distinct sets of data have been reported (*t*-Student test, *P* < 0.05).

## 3. Results and Discussion

### 3.1. Preparation and Characterization of Polyelectrolyte Complexes (PECs)

Dextran (DXS) is a complex, branched glucan composed of chains of varying lengths (from 3 to 2000 kDa). The straight chain consists of *α*-1,6 glycosidic linkages between glucose molecules, while branches begin from *α*-1,3 linkages. DXS is commonly used by microsurgeons to decrease vascular thrombosis. The antithrombotic effect of DXS is mediated through its binding of erythrocytes, platelets, and vascular endothelium, increasing their electronegativity while reducing erythrocyte aggregation and platelet adhesiveness [17]. 

Poly(allylamine), also named PAH, is a cationic polyelectrolyte prepared by the polymerization of allylamine. It can be used in combination with an anionic polyelectrolyte like poly(sodium styrene sulfonate) to form a layer-by-layer (LbL) adsorbed film of negatively and positively charged polymers. Poly(allylamine hydrochloride) has many biomedical applications. The most prominent use of this polyelectrolyte is in the field of cell encapsulation. A layer-by-layer method is used by alternating positively and negatively charged polyelectrolytes to build a barrier between the cell and the harsher outside environment. Upon cell lysis, the capsule of layered polyelectrolytes maintains its structural integrity and can be used for purposes such as drug delivery [7–10]. 

These polyelectrolytes present different chemical features, in particular the DXS is a biocompatible and biodegradable polyelectrolyte while the PAH is biocompatible and not biodegradable polyelectrolyte. In particular, the intracellular degradation of biodegradable polyelectrolyte (DXS) can lead to the release of therapeutic cargo into cells, while the presence of biosynthetic polyelectrolyte can function as reserve system of cargo [7]. 

Polyelectrolyte complexes (PECs) were obtained by mixing in equal volume two polyelectrolyte solutions: DXS-FITC at concentration of 2 mg/mL and PAH at concentration of 1 mg/mL.

**Scheme 1:**
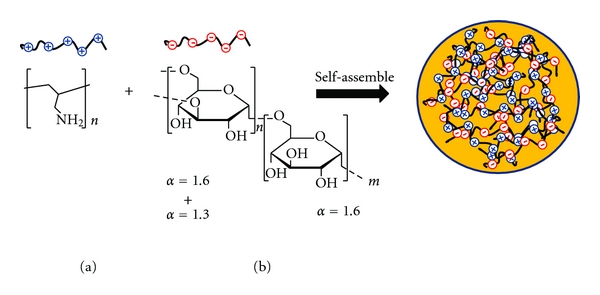
Schematic illustration of the self-assembled poly(allylamine) (a) and dextran (b) polyelectrolyte complexes as drug and gene delivery systems.

DXS is a polyanion, while PAH is polycation and electrostatic attraction occurs between them. In this study, the self-assembly of DXS and PAH was conducted in NaCl solution (pH 6.5). Under this condition, the above two macromolecules having opposite charges were found to self-assemble effectively into PECs. The spontaneous self-assembling of DXS/PAH into PECs was schematically illustrated in Scheme 1. We have assumed that the formation of PECs was a process involved mainly 3 steps. In the first step, the complex formation is mediated by Coulomb forces. At the second step, the formation of new bonds and/or the correction of the distortions inside the polymer's chains may occur within the complexes. Third, an inter-complexes aggregation process may be mediated by the aggregation of secondary complexes *via* hydrophobic interactions. 

### 3.2. Particle Size and *ζ* Potential Measurements

**Figure 1:**
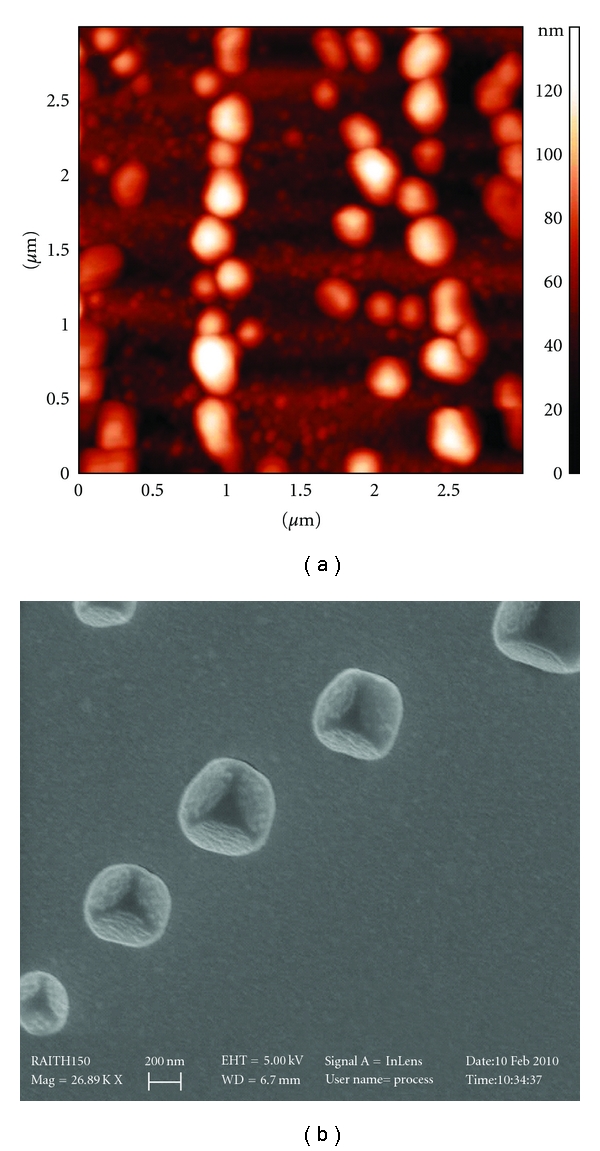
AFM and SEM images of polyelectrolyte complexes ((a) and (b), resp.).

**Figure 2:**
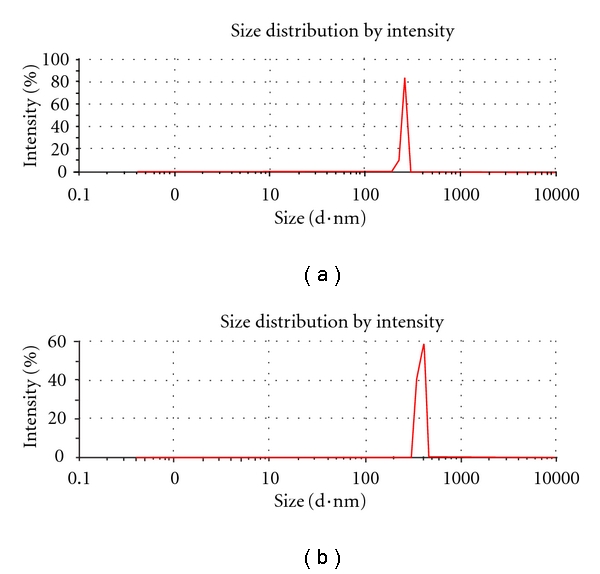
Dynamic light scattering measurements (DLS) of PECs fresh (a) and after 5 days (b).

Dynamic Light Scattering (DLS) determined sizes of PECs resulted in good agreement with SEM and AFM data (Figure 1). In fact, PECs displayed a hydrodynamic diameter of 250 nm (Figure 2(a)). We have also investigated the stability of PECs. Taking PECs during the first two days, particle size was almost unchanged, and even at 5 days, PECs did not aggregate and their size remained lower, around 370 nm (Figure 2(b)). Furthermore, the good stability of PECs supplied an opportunity for storage. 

In addition, the PECs possessed a higher *ζ* potential, around 32 mV. The positive *ζ* potential of PECs made complexes easier to be uptake by cells, attributed to the electrostatic interactions between negative charged cellular membranes and positive charged complexes.

### 3.3. AFM and SEM Characterization

The morphology of PECs was observed by atomic force microscopy (AFM) and scanning electron microscopy (SEM). It is evident from Figure 1 that all the particles have a regular spherical shape and as exhibited in this figure the particle size of the PECs is approximately 250 nm.

### 3.4. Cytotoxicity of PECs

For gene/drug delivery, vectors should not induce cytotoxic effects and the low cytotoxicity of PECs is very important. Here, the cytotoxicity of PECs (DXS/PAH 2 : 1) was evaluated in HeLa cells and BJ cells by MTT test and Trypan Blue assay. We performed cytotoxicity tests at different time points (24, 48 or 72 hours) by using different concentrations of PECs (ranging from 0.01 to 0.2 mg/mL). In particular, it was important to assess cytotoxicity of the nanocomplexes upon 24 hours of incubation since the cells would be in an exponential growth phase during this period and any toxicity that reflects inhibition of proliferation and/or cell death would be clearly visible [18].

Cytotoxicity was evaluated based on cell viability relative to controls as proposed by Kong et al. [19]:

- noncytotoxic: >90% cell viability,
- slightly cytotoxic: 60–90% cell viability,
- moderately cytotoxic: 30–59% cell viability,
- severely cytotoxic: ≤30% cell viability.

**Figure 3:**
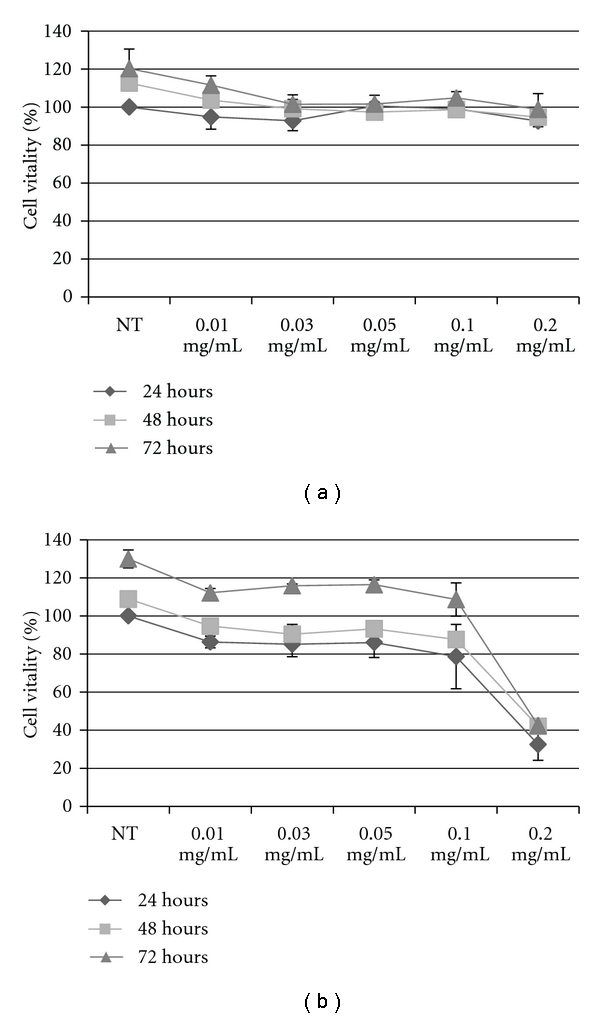
MTT cytovitality test (at 24 to 48 to 72 hours) for the HeLa cells (a) and BJ cells (b), interacting with culture medium (i.e., not treated, NT) and different concentration of PECs. Representative measurements of three distinct sets of data have been reported (*t*-Student test, *P* < 0.05).

**Figure 4:**
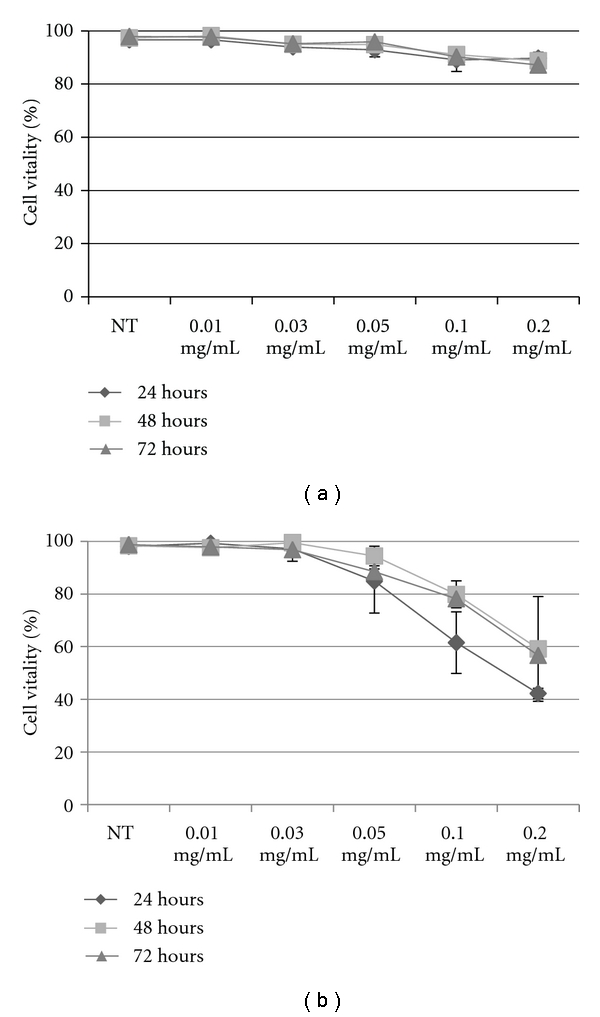
Cell viability assayed by cellular membrane damage assessment using the Trypan Blue exclusion test of PECs in (a) HeLa and (b) BJ cells. Percent cell viability versus PECs concentration for 24 to 48 to 72 hours. Representative measurements of three distinct sets of data have been reported (*t*-Student test, *P* < 0.05).

Figures 3(a) and 4(a) show that cell viability of HeLa cells against PECs was constant on 100% even the concentration was 0.01 mg/mL, 0.03 mg/mL, 0.05 mg/mL, 0.1 mg/mL, and 0.2 mg/mL, showing the much lower cytotoxicity of PECs and therefore the viability of treated cells many nearby to that nontreated cells as control; while, cell viability of BJ cells decreased from 90% to 40% even the concentration was 0.2 mg/mL (Figures 3(b) and 4(b)). The discrepancy in cytotoxicity that has been observed for the two cell lines can be ascribed to the intrinsic characteristics of these cells, since the cell lines used were derived from different cell contexts and expressed differently the molecular machinery, HeLa cells derived from cervix cancer while BJ cell line was established from skin taken from normal foreskin. In order, factors affecting cytotoxicity have been reported to be molecular weight, charge density and type of cationic functionality, structure and sequence (block, random, linear and branched), and conformational flexibility [20].

Since PECs did not impact on metabolism or viability of the two cell lines analyzed, these novel materials could have potential application as safe nanocarriers for gene/drug delivery in neoplastic cells.

### 3.5. In Vitro Uptake

The presence of DXS-FITC allowed the PECs uptake and localization into cancer cells (HeLa) and normal cells (BJ). After 3 hours of incubation with FITC-labelled PECs at concentration of 0.05 mg/mL, HeLa cells and BJ cells were observed by a confocal laser scanning microscope (CLSM).

**Figure 5:**
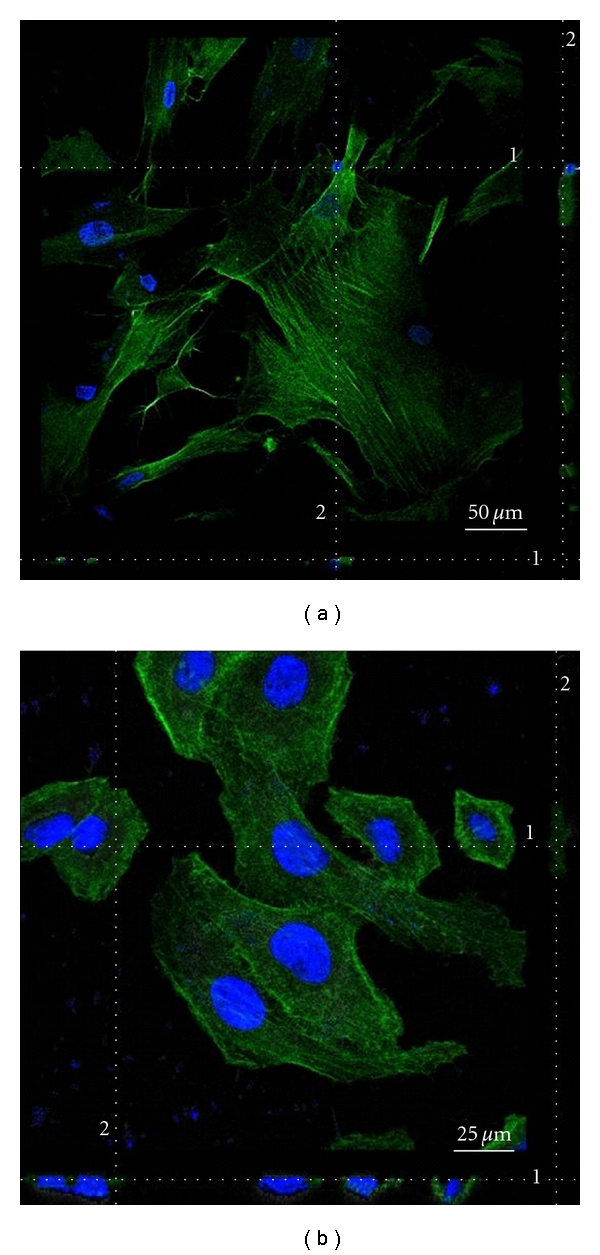
CLSM images of polyelectrolyte complex (PECs) intracellular uptake by (a) human normal fibroblast cells (BJ) and (b) human cervix carcinoma cell line (HeLa). In blue DAPI fluorescence of nuclei. In green FITC fluorescence of PECs. Each confocal image reports the corresponding *Z*-stack optical sections (1, 2) to confirm the PECs internalization. Scale bar: 50 *μ*m (a) and 25 *μ*m (b).

As shown in Figure 5, after 3 hours of incubation, strong green fluorescent staining can be observed, which means PECs have been delivered into HeLa cells and BJ cells. The cell uptake of the PECs was confirmed by performing scans along the Z axis of treated cells, thus showing the predominant localization of the PECs into the cytoplasm of both cell types analyzed. These findings can be explained by electrostatic interactions between the positive surface charge of PECs and the negatively charged cell plasma membrane followed by spontaneous internalisation of PECs inside cells through pinocytosis or endocytosis. This process is also referred to as nonspecific adsorptive endocytosis. The binding of PECs to may be essential for the following process of internalization of the complexes, because the PECs surface was not functionalized for specific binding by cells. Moreover, the FITC-fluorescence signal of PECs, although predominantly associated with sulphated proteoglycan components of the cell plasma membrane, clearly indicated a colocalization of such nanoparticles with actin-*like* cytoskeletal structures or cytosolic endosomal vesicles that are actively involved in their intracellular trafficking and degradability.

## 4. Conclusion

In this study, novel PECs were prepared through an electrostatic interaction between DXS and PAH. These polymeric complexes were spontaneously internalised in normal fibroblast cell line (BJ) and in cervical carcinoma (HeLa) cells, showing a predominant cytoplasmatic localization. More importantly, PECs were herein validated as biocompatible and biodegradable carriers, hence promising gene/drug delivery vehicles at single-cell level.

## References

[1] Jenkins M (2007). *Biomedical Polymers*.

[2] Allen TM, Cullis PR (2004). Drug delivery systems: entering the mainstream. *Science*.

[3] Hughes GA (2005). Nanostructure-mediated drug delivery. *Nanomedicine*.

[4] Panyam J, Labhasetwar V (2003). Biodegradable nanoparticles for drug and gene delivery to cells and tissue. *Advanced Drug Delivery Reviews*.

[5] Kircheis R, Wightman L, Wagner E (2001). Design and gene delivery activity of modified polyethylenimines. *Advanced Drug Delivery Reviews*.

[6] Thomas CE, Ehrhardt A, Kay MA (2003). Progress and problems with the use of viral vectors for gene therapy. *Nature Reviews Genetics*.

[7] Palamà IE, Leporatti S, De Luca E (2010). Imatinib-loaded polyelectrolyte microcapsules for sustained targeting of BCR-ABL^+^ leukemia stem cells. *Nanomedicine*.

[8] De Geest BG, Vandenbroucke RE, Guenther AM (2006). Intracellularly degradable polyelectrolyte microcapsules. *Advanced Materials*.

[9] De Koker S, De Geest BG, Cuvelier C (2007). *In vivo* cellular uptake, degradation, and biocompatibility of polyelectrolyte microcapsules. *Advanced Functional Materials*.

[10] Palamà IE, Coluccia AML, Torre AD (2010). Multilayered polyelectrolyte capsules and coated colloids: cytotoxicity and uptake by cancer cells. *Science of Advanced Materials*.

[11] Ai H, Pink JJ, Shuai X, Boothman DA, Gao J (2005). Interactions between self-assembled polyelectrolyte shells and tumor cells. *Journal of Biomedical Materials Research, Part A*.

[12] Wang C, He C, Tong Z, Liu X, Ren B, Zeng F (2006). Combination of adsorption by porous CaCO_3_ microparticles and encapsulation by polyelectrolyte multilayer films for sustained drug delivery. *International Journal of Pharmaceutics*.

[13] Harada-Shiba M, Yamauchi K, Harada A, Takamisawa I, Shimokado K, Kataoka K (2002). Polyion complex micelles as vectors in gene therapy—pharmacokinetics and in vivo gene transfer. *Gene Therapy*.

[14] Vinogradov S, Batrakova E, Li S, Kabanov A (1999). Polyion complex micelles with protein-modified corona for receptor-mediated delivery of oligonucleotides into cells. *Bioconjugate Chemistry*.

[15] Cheng H, Li YY, Zeng X, Sun YX, Zhang XZ, Zhuo RX (2009). Protamine sulfate/poly(L-aspartic acid) polyionic complexes self-assembled via electrostatic attractions for combined delivery of drug and gene. *Biomaterials*.

[16] Lankalapalli S, Kolapalli VRM (2009). Polyelectrolyte complexes: a review of their applicability in drug delivery technology. *Indian Journal of Pharmaceutical Sciences*.

[17] Lewis SL, Heitkemper MM, Dirksen SR, O’Brien PG, Bucher L (2008). *Medical-Surgical Nursing: Assessment and Management of Clinical Problems*.

[18] Nafee N, Schneider M, Schaefer UF, Lehr CM (2009). Relevance of the colloidal stability of chitosan/PLGA nanoparticles on their cytotoxicity profile. *International Journal of Pharmaceutics*.

[19] Kong N, Jiang T, Zhou Z, Fu J (2009). Cytotoxicity of polymerized resin cements on human dental pulp cells in vitro. *Dental Materials*.

[20] Unger F, Wittmar M, Kissel T (2007). Branched polyesters based on poly[vinyl-3-(dialkylamino)alkylcarbamate-co-vinyl acetate-co-vinyl alcohol]-graft-poly(d,l-lactide-co-glycolide): effects of polymer structure on cytotoxicity. *Biomaterials*.

